# Higher Human Cytomegalovirus (HCMV) Specific IgG Antibody Levels in Plasma Samples from Patients with Metastatic Brain Tumors Are Associated with Longer Survival

**DOI:** 10.3390/medicina59071248

**Published:** 2023-07-05

**Authors:** Inti Peredo-Harvey, Jiri Bartek, Christer Ericsson, Koon-Chu Yaiw, Monica Nistér, Afsar Rahbar, Cecilia Söderberg-Naucler

**Affiliations:** 1 Department of Neurosurgery, Karolinska University Hospital, SE-17176 Stockholm, Sweden; inti.peredo-harvey@regionstockholm.se (I.P.-H.); jiri.bartek@regionstockholm.se (J.B.J.); 2 Department of Medicine Solna, Microbial Pathogenesis Unit, BioClinicum, Karolinska Institutet, SE-17164 Solna, Sweden; koon.chu.yaiw@ki.se; 3 Department of Clinical Neuroscience, Karolinska Institutet, SE-17177 Stockholm, Sweden; 4 Department of Neurosurgery, Rigshospitalet, DK-2100 Copenhagen, Denmark; 5 CSO iCellate Medical AB, SE-17148 Solna, Sweden; christer.ericsson@icellate.se; 6 Division of Neurology, Karolinska University Hospital, SE-17176 Stockholm, Sweden; 7 Department of Oncology-Pathology, BioClinicum, Karolinska Institutet, SE-17164 Solna, Sweden; monica.nister@ki.se; 8 Institute of Biomedicine, Infection and Immunology Unit, MediCity Research Laboratory, Turku University, FI-20014 Turku, Finland

**Keywords:** human cytomegalovirus, brain tumors, immunological response, ELISA

## Abstract

*Background:* Human cytomegalovirus (HCMV) has been detected in tissue samples from patients with glioblastoma but little is known about the systemic immunological response to HCMV in these patients. *Objectives:* To investigate the presence and clinical significance of HCMV antibodies levels in plasma samples obtained from patients with brain tumors. *Materials and Methods:* HCMV-specific IgG and IgM antibody levels were determined in 59 plasma samples collected from brain tumor patients included in a prospective study and in 114 healthy individuals. We examined if the levels of HCMV specific antibodies varied in patients with different brain tumor diagnoses compared to healthy individuals, and if antibody levels were predictive for survival time. *Results*: HCMV specific IgG antibodies were detected by ELISA in 80% and 89% of patients with GBM and astrocytoma grades II–III, respectively, in all samples (100%) from patients with secondary GBM and brain metastases, as well as in 80% of healthy donors (*n* = 114). All plasma samples were negative for HCMV-IgM. Patients with brain metastases who had higher plasma HCMV-IgG titers had longer survival times (*p* = 0.03). *Conclusions*: HCMV specific IgG titers were higher among all brain tumor patient groups compared with healthy donors, except for patients with secondary GBM. Higher HCMV specific IgG levels in patients with brain metastases but not in patients with primary brain tumors were associated with prolonged survival time.

## 1. Introduction

Current knowledge of the etiology of brain tumors remains fragmentary and the prognosis for affected patients is very poor. Important progress was made towards understanding the pathogenesis of brain tumors, when it was discovered that human cytomegalovirus (HCMV) is present in a majority of brain tumors such as glioblastoma (GBM), medulloblastoma and metastatic brain tumors [1,2,3,4,5] and that treatments acting against this virus indicate highly improved survival time in patients with glioblastoma [6,7,8]. The relevance of these findings was early questioned, as some investigators found evidence of HCMV nucleic acids and proteins in a majority of these tumors or in the patient’s blood [9,10,11,12,13,14,15,16,17,18,19], while others failed to detect this virus in brain tumor patient´s specimens [20,21,22,23,24,25,26,27]. The use of sensitive and specific techniques for virus detection in tumors potentially resolves this apparent paradox, as differences in obtained results appear to be caused by technical aspects of methods used for virus protein detection [28], such as the use of antigen retrieval protocols. Detection of virus nucleic acids in glioblastoma tissue samples by PCR or sequencing methods is however also difficult [20,21,26,29,30], although some investigators have detected HCMV DNA/RNA in glioblastoma tumors using PCR [1,11,31], or in situ hybridization techniques [19].

It is not known why optimized techniques are needed for the detection of HCMV proteins in tumor tissue specimens, as this virus is easily detected in tissue specimens obtained from patients with acute HCMV infections. Viral load differences may provide some explanations, but clearly, other factors are at play. This may relate to the fact that certain unique HCMV strains appear to be present in tumors; these viruses exhibit altered growth behaviors in vitro [32,33], and this could possibly explain the difficulties in the ability to detect them in tumors. These observations are highly concerning as they suggest that certain HCMV strains with unique features are associated with tumors and these may induce oncogenic transformation and initiate cancer development, while most HCMV strains would not have this capacity.

HCMV is a β-herpesvirus with very high prevalence in humans (80–100%). The primary infection is generally asymptomatic or mild and leads to lifelong latent/persistent infection, from which it can reactivate. While considered harmless in most immunocompetent individuals, HCMV can cause major morbidity and mortality among immunosuppressed people, such as transplant patients and AIDS patients. It is also the most common congenital infection and can lead to life-long sequelae in infants infected with HCMV. Although the immune response to HCMV is important to determine the outcome of the infection, a previous HCMV infection does not protect against re-infection. About 30% of children who developed hearing loss due to a congenital infection were found to be born from HCMV seropositive mothers [34]. Similar observations have been made in transplant patients; patients who have had an earlier HCMV infection were not protected from infection with another strain and multiple strains were found in the same individuals. Hence, one individual can be infected multiple times with different HCMV strains during their life, some of which may exhibit oncogenic capacity.

After a primary infection, HCMV establishes latency primarily in CD34-positive progenitor cells in the bone marrow. Terminal differentiation of monocytes into macrophages or dendritic cells, induced by inflammatory cytokines, can result in the reactivation of latent viruses [35,36]. Once reactivated, HCMV is capable of spreading to other cells, and through sophisticated mechanisms, this virus can control numerous cellular pathways, which are relevant for understanding its potential role in cancer.

A number of previous studies have reported a high prevalence of HCMV proteins in brain tumor specimens [2,12,37], as well as in a majority of colon, breast, prostate, and ovarian cancer specimens [38,39,40,41]. We also found HCMV in over 90% of brain metastases originating from colon and breast cancer [42]. As healthy tissue surrounding the primary tumor or metastases is virus negative, the question arises, does HCMV play a role in the establishment or progression of these tumors or does it merely represent an epiphenomenon? We therefore examined whether the viral load in GBM tumors at the time of the patient´s diagnosis affected the prognosis for these patients. We found that patients with lower virus levels in their tumors at diagnosis survived significantly longer than those with high viral activity [16], which implies a potential role of HCMV in tumor progression. This hypothesis is supported by numerous reports demonstrating that HCMV can promote tumor growth [43,44,45,46]. HCMV exerts various oncomodulatory roles in tumors [3,4,5,6,7,8,9,10,40,41,42,43,47,48] and can in fact establish all the ten Hallmarks of Cancer. This virus controls cellular gene expression via direct and epigenetic mechanisms, it causes DNA damage and mutations, it controls cell cycle progression and changes cellular metabolism to promote cell growth. HCMV also induces inflammation and angiogenesis, and controls immune functions to avoid detection and elimination by the immune system [45]. Most HCMV strains cause a lytic infection that results in cell death, and it is unlikely that such strains can cause cancer. However, the slow-growing HCMV strains that have been retrieved from breast cancer and glioblastoma tumor specimens replicate slowly in culture and do not appear to cause cell death. This may be a dangerous feature of cancer-associated HCMV strains. It is also possible that HCMVs ability to affect tumor cells without causing a lytic infection may be another possible way to explain its association with cancer.

Little is known about the general immune control of HCMV in brain tumor patients. During brain radiation therapy, 48% of brain tumor patients reactivate HCMV, of which many develop encephalitis-like symptoms and have a very poor prognosis [46]. As the immune response is important to control HCMV infections in immunocompromised patients, it is possible that immune control of HCMV reactivations also plays an important role in the risk of reactivating HCMV during radiation therapy. It is therefore important to learn more about the immune systems role in controlling HCMV in brain tumor patients, as such information could aid in the development of immune-based treatment protocols for these patients. In the present study, we determined the levels of HCMV-specific antibodies in plasma samples from 59 brain tumor patients collected in a prospective trial and 114 in healthy blood donors and examined if the HCMV-specific antibody levels affected the patient outcome.

## 2. Materials and Methods

### Clinical Samples

Plasma samples were collected in a prospective study at the time of surgery from 59 patients operated for different types of brain tumors at the Karolinska University Hospital (between 2005 and 2009), to be evaluated for HCMV serology. The patients had the following diagnoses: glioblastoma WHO grade IV (GBM) (*n* = 25), astrocytoma grades II–III (*n* = 18), secondary GBM (*n* = 4), ganglioglioma (*n* = 1) and meningioma (*n* = 1) and brain metastasis (*n* = 10, primary tumors were ovarian cancer (*n* = 1), breast cancer (*n* = 4), malignant melanoma (*n* = 1), non-small cell lung cancer (*n* = 4) (Table 1). Information about the treatment of these patients can be found in Supplementary Table S1. Plasma samples from 114 healthy donors (age range 20–69 years) were obtained in 2011 from the blood center at our hospital. This study was registered and approved by Stockholm’s ethics committee (2005/542-31/1, 1 June 2005 and 2006/755-31-2, 5 July 2006) and by the ethics committee at the Karolinska Institute (2008/628-31/2, 22 May 2008, 2008/518-31, 18 June 2008 and 2014/2154-32, 16 February 2015). Written informed consent was obtained from all subjects involved in this study.

## 3. HCMV Serology

Plasma samples stored at −80 °C were analyzed for HCMV-specific IgG and IgM with commercially available kits (Enzygnost Anti-CMV IgG and IgM separate kits, Siemens Healthcare Diagnostics Products, Erlangen, Germany) according to manufactory instruction. Plasma samples were diluted and assayed in duplicate in 96-well plates coated with HCMV/control antigens. Conjugated anti-human IgG/IgM antibodies and substrate solution were used for the detection of HCMV-specific IgG/IgM antibodies. Internal positive and negative controls (included in the kit) were used in each plate. According to the manufacturer’s instructions, a cut-off value of Optical density (OD) is set to ≥0.2 for a positive sample for both HCMV-specific IgG and IgM.

### Statistical Analysis

Results were expressed as mean ± standard deviation mean (SDM) and analyzed with an Unpaired student *t*-test. The median times to endpoints were estimated from the Kaplan–Meier curves, with 95% CIs. Analysis was performed using Graph Pad Prism software version 9. *p* < 0.05 was considered significant.

## 4. Results

**HCMV specific IgG antibody levels in plasma are higher in brain tumor patients as compared to age-matched healthy blood donors.**

**Figure 1:**
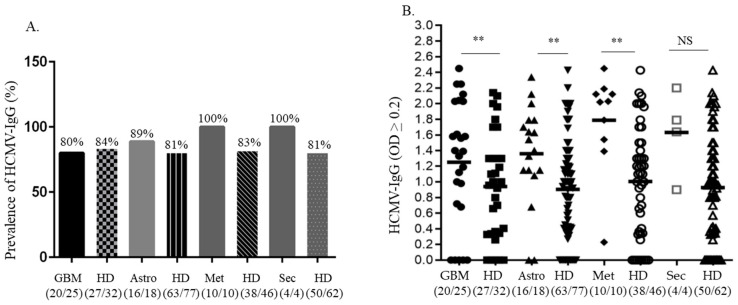
Prevalence and HCMV specific IgG titers in plasma samples obtained from patients with different types of brain tumors. A high prevalence of HCMV specific IgG was detected in patients with metastatic brain tumors and in the few patients with secondary GBM compared to age-matched healthy blood donors, but not in the larger group of glioblastoma patients (**A**). HCMV specific IgG titers were significantly higher in plasma samples from patients with GBM (**; *p* = 0.008), astrocytoma grades II–III (**; *p* = 0.003) and brain metastasis (**; *p* = 0.004) as compared to age-matched healthy blood donors (**B**). The few patients with secondary GBM had higher mean OD values than controls, but this was not significant (NS; *p* = 0.16), likely due to the low sample numbers. Cut off value of OD: ≥0.2 was considered positive (panel B). Unpaired student *t*-test was used for the evaluation of HCMV specific IgG levels in each cohort of patients compared with age-matched healthy donors. GBM; Glioblastoma, Astro; astrocytoma grades II–III, Met; Metastatic brain tumor, Sec; secondary GBM, HD; healthy blood donors.

HCMV specific IgG was detected in 20/25 (80%) plasma samples obtained from GBM patients, in plasma from 16/18 (89%) patients with astrocytoma grades II–III, and in plasma from all patients with brain metastasis and secondary GBM (Figure 1A,B). The prevalence of HCMV-IgG was 80% (91 of 114 were positive) in healthy blood donors. The IgG levels were higher in patients with GBM (*p* = 0.008), astrocytoma grades II–III (*p* = 0.003) and brain metastases (*p* = 0.004), respectively, compared to age-matched healthy donors but were not significantly higher in the few patients with secondary GBM (*p* = 0.16) (Figure 1B). In addition, one patient with ganglioglioma had an OD value equal to 1.2 and one patient with meningioma had an OD equal to 1.4. A cut-off value of OD: ≥0.2 was considered positive for HCMV-specific IgG and IgM, respectively (according to the kit). All patients and healthy donors were negative for HCMV-IgM.

**Patients with metastatic brain tumors had higher HCMV specific IgG levels and prolonged median overall survival time.**

**Figure 2:**
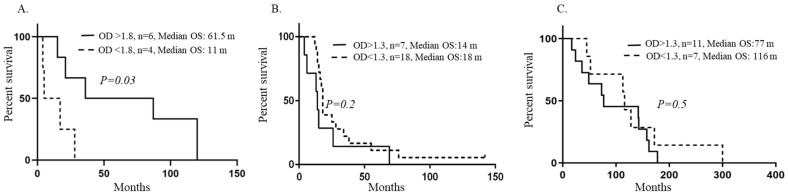
Significance of HCMV specific IgG levels on patients’ overall survival. Patients with brain metastases who had higher plasma HCMV specific IgG levels lived significantly longer after primary cancer diagnosis compared with those with lower HCMV specific antibody levels (**A**). Median overall survival did not differ in GBM patients (**B**) or in patients with astrocytoma grades II–III (**C**) who had high versus low HCMV specific IgG levels. The median overall survival times to endpoints were estimated from the Kaplan–Meier curves, with 95% CIs.

We next examined if HCMV specific IgG levels were associated with patient outcomes. We compared the outcome of patients who had over or below mean levels of antibodies for each cohort. We found that patients with brain metastases who had HCMV-specific IgG levels with a mean value over 1.8 (OD > 1.8), had longer overall survival (OS) time after primary cancer diagnosis compared with those with lower antibody levels (*p* = 0.03, OS: 61.5, all female, mean age: 51.8 years with interval: 27–71 years vs. OS: 11 months (all male, mean age: 59 years with interval: 53–65 years) (Figure 2A and more detailed information about this cohort of patients can be found in Supplementary Table S2). In this cohort of patients, median OS also trended longer in patients with higher HCMV specific IgG levels (OD > 1.8) after diagnosis of brain metastasis compared with those with lower antibody levels, but did not reach statistical significance (*p* = 0.09, OS: 10 vs. 3.5 months). The median overall survival time did not differ in patients with glioblastoma (Figure 2B) or astrocytoma (Figure 2C), and was not associated with higher or lower HCMV-specific IgG levels. Too few patients with secondary glioblastoma were available for survival analyses. The patient with ganglioglioma survived 127 months and the patient with meningioma survived 146 months (Table 1).

## 5. Discussion

In this study, we examined the prevalence and levels of IgG and IgM in plasma samples from brain tumor patients and controls and evaluated the potential significance of HCMV-specific IgG levels on the survival of brain tumor patients. HCMV-IgG was present in 52 of 59 patients with brain tumors, (including one patient with ganglioglioma and one patient with meningioma), which represents 88% of the cases. Among 114 healthy blood donors, 80% were HCMV-IgG positive. HCMV specific IgG levels were significantly higher in brain tumor patients compared to corresponding age-matched healthy blood donors. Although this patient cohort represents a small sample size, the HCMV-specific IgG prevalence observed in our study is comparable to published data [49]. The plasma samples included in this sample cohort were collected in a prospective study from 2005–2009, while samples from healthy blood donors were collected in 2011. The plasma samples were collected and prepared with the same methods and the samples were then run at the same time in the ELISA assay. It can however not be excluded that the timing of sample collection could have affected these data, as some evidence suggests that HCMV epidemics come in different time intervals.

We observed that a higher IgG response to HCMV was associated with better outcomes in patients diagnosed with brain metastases, but this was not seen in patients with a primary brain tumor diagnosis. Patients with metastatic brain tumors (primary tumors: ovarian cancer (*n* = 1), breast cancer (*n* = 4), malignant melanoma (*n* = 1), who had HCMV-specific IgG levels over a mean OD value of 1.8 for the cohort (OD > 1.8) had longer overall survival (OS) time after primary cancer diagnosis compared to patients (non-small cell lung cancer adenocarcinoma, *n* = 4) who had lower antibody levels (median OS:61.5 vs. 11 months, *p* = 0.03). Thus, it is possible that lower antibody titers reflect a less active immune response to HCMV among patients with brain metastases and that this may predict a poor outcome, but the primary diagnosis may also influence this interpretation. Thus, patients who respond to emerging HCMV activity with higher HCMV-specific antibody titers, and possibly also with a stronger T cell response towards HCMV may have a better outcome. We earlier reported that a higher abundance of HCMV proteins in brain metastases of colon cancer and breast cancer patients was associated with worse outcomes [42]. Those patients who have a lower immune control of HCMV, potentially depicted by lower HCMV specific IgG levels, may hence have higher viral activity in their tumors and a worse outcome.

The sample size of patients with brain metastases was small in the present study, and studies of larger patient cohorts are needed to further confirm the significance of HCMV specific IgG levels in the clinical outcome of patients with brain metastases. Future tests should preferably also utilize different ELISA tests for parallel sample evaluation to ascertain that the data is not affected by certain tests used. Nevertheless, the results are interesting and should stimulate others to investigate this potential association further. We did not observe a difference in survival rates in patients with GBM or astrocytoma who had low or high antibody levels (OD values) in this small number of patients. Of note, 9 of 25 GBM patients, 2 of 18 patients with astrocytoma grades II–III, and 2 of four patients with secondary GBM received treatment with the antiviral drug Valganciclovir in addition to their standard therapy. In the present study, we cannot exclude the possibility that antiviral treatment affected the immunological responses or patient outcome, in particular in the case of a possible HCMV reactivation. Nine GBM patients who were treated with Valganciclovir lived 9.5 months longer than those without antiviral therapy (median OS of 26 months vs. 16.6 months, respectively). This is in line with our previously published treatment data [7]. One GBM patient treated with Valganciclovir was alive at the time of study closure (OS: 142 months).

Other investigators have also reported a worse outcome in brain tumor patients with a lower HCMV antibody response. In a study including 362 cases, higher levels of HCMV-IgG were associated with decreased glioma risk; those with the lowest IgG levels had the highest risk. In a study of 374 brain tumor patients (glioblastoma, astrocytoma, oligodendroglioma/oligoastrocytoma, and brain metastases), anti-HCMV-pp65 IgG titers were significantly lower than in healthy donors. Patients with a weaker anti-HCMV-IgG response had a decreased median overall survival time (419 vs. 667 days, *p* = 0.017) [50]. Others reported that HCMV-IgG positive GBM patients had shorter survival than HCMV- IgG negative patients [49], but the impact of titers was not examined. We earlier observed higher HCMV-IgG titers among GBM patients than controls when using HCMV AD169 strain-derived antigens for the ELISA test. We found that those with higher antibody titers had an increased median overall survival time compared to those with lower IgG titers [42]. We now made similar observations among patients with brain metastases, but not in GBM patients. It is possible that the immune response to certain antigens has a more relevant impact on virus control, but different results may also depend on the test used. Under poor control of virus activity, HCMV could through its numerous mechanisms affecting cellular and immunological functions providing ways to create a more aggressive tumor that is less responsive to currently used therapies, and this would affect the patient’s prognosis. This merits further investigation, as if confirmed true, antiviral therapy could lower HCMV activity in brain tumor patients and possibly improve their currently poor response to therapy and improve their poor prognosis.

If the virus plays an important role in cancer, HCMV may represent a novel target for therapy. In animal models, antiviral treatment against HCMV or nanobodies to HCMV US28 was shown to prevent the growth of HCMV-positive human medulloblastoma, neuroblastoma, and GBM xenograft tumors [51,52,53]. Antiviral treatment of HCMV or immunotherapy targeting HCMV in glioblastoma patients has also indicated promising results of unexpected substantially prolonged patient survival time [6,7]. We reported that treatment of patients with GBM with the antiviral drug Valganciclovir as add on to standard therapy is associated with prolonged median overall survival (OS 24.1 months for 102 Valganciclovir treated patients, versus 13.3 months in 231 contemporary controls, *p* < 0.0001) [7]). Other investigators have shown that improving HCMVs immune response in glioblastoma patients with dendritic cell vaccination is also associated with improved outcomes. In sharp contrast, other medical and immunotherapy protocols have failed to affect the prognosis of these patients [54]. Taken together, it is possible that HCMV affects tumor progression in glioblastoma patients and in other brain tumor patients as well, and that HCMV-targeted therapies could improve the prognosis for these patients. This would be highly warranted, as other medical therapies have so far not shown any improvement in the survival chances for these patients [54]. Ongoing randomized clinical trials are currently evaluating whether antiviral treatment to HCMV or HCMV dendritic cell vaccination with pp65 mRNA in glioblastoma patients has a place in the treatment of these patients (NCT04116411, NCT03927222).

## 6. Conclusions

We found that HCMV specific IgG levels were higher in brain tumor patients than in age-matched healthy blood donors, which implies a higher HCMV activity in these patients than in controls. Higher HCMV-IgG levels were associated with longer survival in patients with brain metastases, but not in patients with primary brain tumors such as glioblastoma or astrocytoma. These observations suggest that the immune response to HCMV in brain tumor patients should be further investigated in clinical trials along with studies assessing whether antiviral therapy to HCMV or immunotherapy targeting HCMV can influence the currently negative treatment results for brain tumor patients.

## Figures and Tables

**Table 1:** Patient’s characteristics.

Diagnosis	Gender M F (%)	Age (Median, Y) (Interval)	MIB Index (Interval)	OS (Median) (m)	Alive (n)
Primary GBM (*n* = 25)	20 5 (80) (20)	61 (44–74)	10–60	18	1
Astrocytoma (Grades II–III, *n* = 18)	11 7 (61) (39)	44 (23–58)	5–40	114.5	4
Brain Metastasis * (*n* = 10)	4 6 (40) (60)	59 (27–70)	ND	5	0
Secondary GBM (*n* = 4)	2 2 (50) (50)	53.5 (29–73)	15–100	76.5	0
Ganglioglioma (Grade 1) (*n* = 1)	1 -	20	ND	127	1
Meningioma (*n* = 1)	- 1	16	ND	146	1

*n*; numbers, *; ovarian cancer (*n* = 1), breast cancer (*n* = 4), malignant melanoma (*n* = 1), non-small cell lung cancer (*n* = 4). Y; years, m; months, OS: Overall survival, ND: not done.

## Data Availability

Data will be available upon request.

## Supplementary Materials

The following supporting information can be downloaded at: https://www.mdpi.com/article/10.3390/medicina59071248/s1, Table S1: Information on treatment of patients with GBM, Secondary GBM, Astrocytoma (Grades II–III), Brain Metastasis, Gangliogliom WHO I and Meningeom WHO I. Table S2: Detailed information on patients with brain metastases.
