# Supplementary material for: Higher Human Cytomegalovirus (HCMV) Specific IgG Antibody Levels in Plasma Samples from Patients with Metastatic Brain Tumors Are Associated with Longer Survival
Source: Medicina (Kaunas). 2023 Jul 5;59(7):1248. doi: 10.3390/medicina59071248 (PMC10384986; doi:10.3390/medicina59071248)
Supplement: Supplementary file 1 [file medicina-59-01248-s001.zip › medicina-2415707-supplementary.pdf]

Supplementary Table S1. Information on treatment of patients with GBM, Secondary GBM, Astrocytoma (Grades II-III), Brain Metastasis, Gangliogliom WHO I and Meningeom WHO I.

**Secondary GBM**	**Treatment**
1	GTR, TMZ, VGV
2	GTR, Reop, CCNU, TMZ, Vepecid
3	Biopsia, RT, CCNU, GTR,TMZ
4	PR, RT, GTR, CCNU, TMZ, VGV

**Astrocytoma (Grades II-III)**	**Treatment**
1	GTR, CCNU, GN, Reop, RT, TMZ, Avastin
2	PR, RT, CCNU, GNx2, Reop, TMZ
3	GTR, RT, Reop, CCNU
4	Biopsia, RT, GTR,TMZ, Reop, CCNU

5	GTR, RT
6	GTR, RT, TMZ, Reop, RT
7	GTR, RT
8	PR, RT, CCNU, GNx2, Reop, TMZ
9	PR, RT, CCNU, Reop, TMZ
10	GTR, RT, CCNU, Vepesid
11	GTR, TMZ, RT+TMZ, VGV
12	GTR, CCNU, GN, TMZ
13	PR, RT, CCNU, RT, TMZ, CCNU, Avastin, Vepesid
14	GTR, RT, CCNU, Reop, TMZ, Avastin
15	GTR, RT, TMZ, RT, CCNU, VGV, Avastin
16	PR, RT
17	PR, RT, Reop, GN, TMZ, CCNU
18	GTR, RT

**Brain Metastasis**	**Treatment**
1	GTR
2	GTR
3	GTR
4	GTR
5	GTR
6	GTR

7	GTR
8	GTR
9	GTR
10	GTR

Benign	Treatment
Gangliogliom WHO I	GTR
Meningeom WHO I	GTR

PR: partiell resection, GTR: Gross Total Resection, RT: radio therapy

TMZ: temozolamide, CCNU: Lomustine, VGV: valganciclovir,

Reop: reoperation, GN: GammaKnife, Natulan: Procarbamazepine,

Vepesid: etoposide

Supplementary Table S2. Detailed information on patients with brain metastases.

Patients with brain metastasis	HCMV specific IgG (optical	Primary diagnosis	Gender	Age	OS after diagnosis for brain metastasis (months)	Treatment (primary tumor)	OS after primary cancer diagnosis (months)	Treatment (brain metastesis)

	**density values)**							
1	> 1.8	ovarian cancer	Female	27	5	Gross Total Resection	15	Local surgery, chemotherapy.
2	>1.8	Breast cancer	Female	49	24	Gross Total Resection	120	Surgery, radiotherapy and chemotherapy, new surgery of breast cancer. Gamma knife treatment of brain metastasis, surgery of one brain metastasis.
3	>1.8	Malignant melanoma	Female	50	16	Gross Total Resection	120	Surgery of skin melanoma, surgery another skin melanoma. Lung and brain metastasis, Temozolomide, surgery brain metastasis, gamma knife treatment, second gamma knife treatment. Surgery of new brain metastasis, third gamma knife treatment.
4	>1.8	Breast cancer	Female	69	2	Gross Total Resection	21	Adjuvant chemotherapy, local radiotherapy, brain metastasis surgery.
5	>1.8	Breast cancer	Female	70	4	Gross Total Resection	36	Surgery, local radiotherapy, chemotherapy. Gamma knife treatment of brain metastasis 2 times, surgery of recurrence/radio necrosis.

6	>1.8	Breast cancer	Female	71	15	Gross Total Resection	87	Surgery, chemotherapy, hormone treatment. Chemo against bone metastasis and liver metastasis. Surgery of one metastasis and gamma knife treatment three times of other metastasis.
7	< 1.8	Non-small cell lung cancer	Male	53	5	Gross Total Resection, radio therapy	5	Surgery of brain metastasis and radiotherapy (whole brain) and for other brain metastasis.
8	< 1.8	Non-small cell lung cancer	Male	54	3	Gross Total Resection, local radio therapy	4	Chemotherapy, surgery of brain metastasis, post-surgery, and radiotherapy.
9	< 1.8	Non-small cell lung cancer	Male	64	0	Gross Total Resection	17	Local radiotherapy and chemotherapy. Gamma knife treatment of brain metastasis. Resection of same metastasis.
10	< 1.8	Non-small cell lung cancer	Male	65	4	Gross Total Resection	28	Local surgery, local radiotherapy and chemotherapy. Gamma knife treatment of metastasis, surgery of 1 recurrence.
